# Infant mortality in Italy: large geographic and ethnic inequalities

**DOI:** 10.1186/s13052-023-01571-z

**Published:** 2024-01-17

**Authors:** Silvia Simeoni, Luisa Frova, Mario De Curtis

**Affiliations:** 1 ISTAT, Direzione Centrale Per Le Statistiche Sociali E Il Welfare, Servizio Sistema Integrato Salute, Assistenza e Previdenza, Istituto Nazionale Di Statistica, Rome, Italy; 2 Dipartimento Materno Infantile, Università Degli Studi Di Roma La Sapienza, Rome, Italy

**Keywords:** Neonatal mortality, Post-neonatal mortality, Infant mortality, Ethnic inequalities, Geographical inequalities, Covid-19, Poisson regression model

## Abstract

**Background:**

Neonatal and infant mortality rates are among the most significant indicators for assessing a country's healthcare and social development.

This study examined the trends in neonatal, post-neonatal, and infant mortality in Italy from 2016 to 2020 and analysed differences between children of Italian and foreign parents based on areas of residence, as well as the leading causes of death. Special attention was given to the analysis of mortality in 2020, the first year of the Covid-19 pandemic, and its comparison with previous years.

**Methods:**

Data from 2016 to 2020 were collected by the Italian National Institute of Statistics and extracted from two national databases, the Causes of Death register and Live births registered in the population register. Neonatal, post-neonatal, and infant mortality rates were calculated using conventional definitions. The main analyses were conducted by comparing Italian citizens to foreigners and contrasting residents of the North with those of the South. Group comparisons were made using mortality rate ratios. The main causes of death were examined, and Poisson log-linear regression models were employed to investigate the relationships between mortality rate ratios for each cause of death and citizenship, place of residence and calendar year.

**Results:**

In Italy, in 2020, the neonatal mortality rate was 1.76 deaths per thousand live births and it was 55% higher in foreign children than in Italian children. Foreign children had a higher mortality rate than Italians for almost all significant causes of death. Children born in the South of Italy, both Italian and foreign, had an infant mortality rate about 70% higher than residents in the North. Regions with higher infant mortality were Calabria, Sicily, Campania, and Apulia. In the South, mortality from neonatal respiratory distress and prematurity was higher.

In the first months of 2020, between March and June, the first Covid-19 wave, Italy experienced an increase in neonatal and infant mortality compared to the same period in 2016–2019, not directly related to SARS-CoV-19 infection. The primary cause was neonatal respiratory distress.

**Conclusions:**

The neonatal and infant mortality rates indicate the persistence of profound inequalities in Italy between the North and the South and between Italian and foreign children.

**Supplementary Information:**

The online version contains supplementary material available at 10.1186/s13052-023-01571-z.

## Background

Neonatal and infant mortality rates are among the most significant indicators for assessing a country's health and civil development, and their monitoring is an important tool for tracking changes over time that can be influenced by healthcare, social, economic, and organizational factors [1–3]. Emphasizing the global importance of these indicators is their inclusion in the Sustainable Development Goals (SDGs) adopted by all UN member states. Specifically, SDG target 3.2 strives to reduce the neonatal mortality rate (NMR) and eliminate all preventable deaths of newborns by 2030 [4].

The economic crisis that began in 2008 and the progressive cuts in funding to the Italian Health Service by all governments in recent years are a cause for concern regarding the maintenance of care levels for all stages of life, including in the maternal and child health field [5, 6]. Monitoring changes in neonatal and infant mortality over time is essential for predicting and implementing effective social and healthcare policies aimed at reducing inequalities.

In a previous study, we observed higher neonatal and infant mortality in the southern regions and among children of foreign parents from 2006 to 2015 [7]. The study aimed to evaluate the trends in neonatal and infant mortality from 2016 to 2020 and analyse geographical differences in mortality among children of Italian and foreign parents, the leading causes of death, and potential epidemiological changes during the first year of the Covid-19 pandemic.

## Materials and methods

This study presents the most recent data on neonatal and infant mortality in Italy, sourced from the Italian National Institute of Statistics (ISTAT). The data were obtained from two national databases, the Causes of Death register and Live births registered in the population register.

The Causes of Death register includes all the deaths that occurred on the Italian territory in a calendar year, for which causes of death and demo-social information are reported. Data are collected through the death certificates [8]. The cause of death coding as well as the selection of the underlying cause of death are based on the ICD-10 rules (International Statistical Classification of Diseases, Injuries and Causes of Death, X Revision) [9].

The Live births registered in the population register collects the number of live births residing in Italy in a calendar year. This information is derived from the birth registration models in the population register, acquired through notifications sent by Italian municipalities to the ANPR (National Register of the Resident Population) system. In this register, demo-social information, such as citizenship, is reported [10].

According to standard definitions, neonatal mortality indicates the number of deaths within the first 28 days of life per thousand live births, post-neonatal mortality is the number of deaths between 28 days and the end of the first year, and infant mortality is the number of deaths in the first year of life per thousand live births.

The study reports neonatal, post-neonatal, and infant mortality rates (NMR, PMR and IMR, respectively) for the period from 2016 to 2020. For the year 2020, these rates have been presented for the regions with higher birth rates (more than 10,000 births per year) to avoid errors associated with small numbers. An analysis of infant mortality was also conducted in five areas: North West (Piedmont, Aosta Valley, Lombardy, Liguria), North East (Veneto, Trentino-South Tyrol, Friuli Venezia Giulia, Emilia-Romagna), Central (Tuscany, Marche, Umbria, Lazio), Southern regions (Abruzzo, Molise, Campania, Basilicata, Calabria, Apulia), and Islands (Sardinia and Sicily). Some data were referred to the North (including North West and North East) and to the South (including Southern and Island regions).

A detailed analysis of neonatal, post-neonatal, and infant mortality was conducted concerning parents' citizenship and area of residence. Children with at least one parent holding Italian citizenship were classified as Italian, while those with non-Italian citizenship were considered foreign. The Mortality Rate Ratio (MRR), the ratio between two different rates and confidence intervals at 95% (CI95%) were calculated to assess significant differences in mortality between Italians and foreigners, regions, and geographic areas.

Variations in the causes of death from 2016–2019 to 2020, the first year of the Covid-19 pandemic, were studied considering area of residence. MRR and confidence intervals (CI95%) were calculated.

The relationships between mortality rate ratios (MRR) for each cause of death and the independent variables as gender, age (differentiated into neonatal and post-neonatal age), residence, citizenship, and calendar year were estimated using Poisson log-linear regression models. This model enables us to estimate how each independent variable influences the MRR while taking into account the impact of the other independent variables. The independent variables were studied simultaneously in the model, allowing us to determine which of these had the most influence on the dependent variable [11].

## Results

**Fig. 1:**
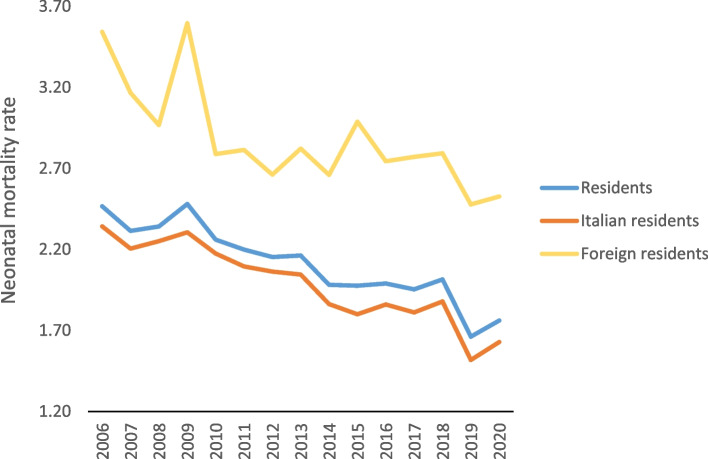
Neonatal mortality rates by citizenship, per thousand live births residing in Italy

In 2020, 404,892 babies were born, of which 345,100 (85.2%) had Italian parents and 59,792 (14.8%) had foreign parents [6]. In 2020, there were 713 neonatal deaths before the 28th day of life, with 562 occurring in children of Italian parents and 151 in children of foreign parents. The total neonatal mortality (70.2% of infant mortality) decreased from 1,99 per thousand live births in 2016 to 1.66 in 2019, but in 2020, there was a halt, with a rate of 1.76 (Fig. 1).

The rate was 1.63 per thousand for children of Italian parents and 2.53 per thousand for children of foreign parents (MRR foreigners/Italians = 1.55, meaning foreign infants had a 55% higher mortality rate than Italian infants).

**Fig. 2:**
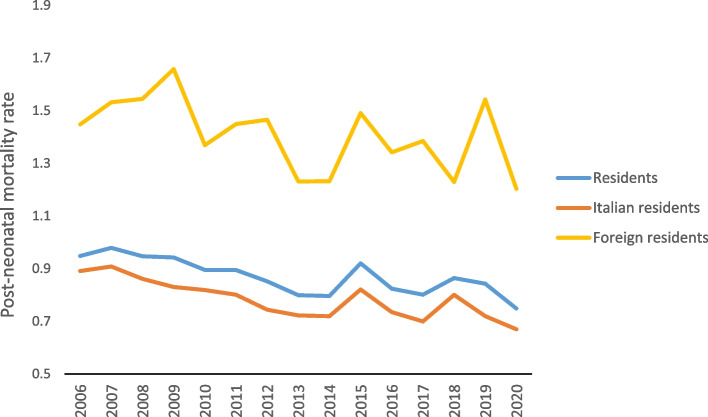
Post-neonatal mortality rates by citizenship, per thousand live births residing in Italy

In 2020, a total of 303 children died between the 28th day of life and the first year, with 231 being children of Italian parents and 72 being children of foreign parents. The post-neonatal mortality rate was 0.75 per thousand: 0.67 per thousand for children of Italian parents and 1.20 per thousand for children of foreign parents (MRR foreigners/Italians = 1.80, meaning foreign infants had an 80% higher mortality rate). Post-neonatal mortality accounted for 29.8% of the total infant mortality. Unlike neonatal mortality, post-neonatal mortality decreased in 2020 for both Italian and foreign children residing in Italy (Fig. 2).

**Fig. 3:**
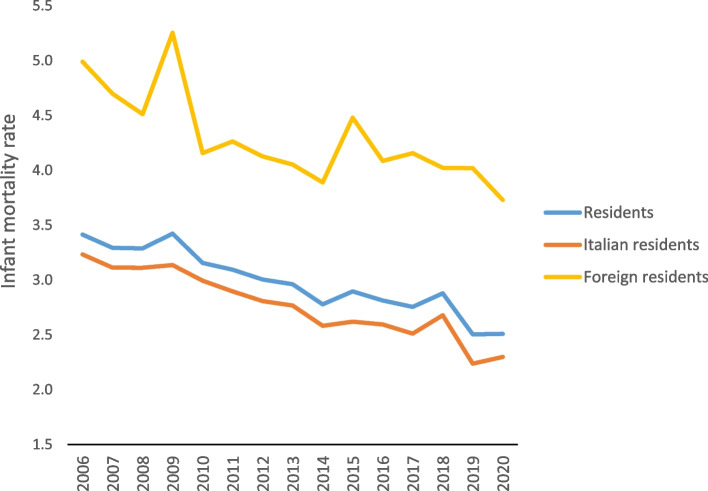
Infant mortality rates by citizenship, per thousand live births residing in Italy

In 2020, a total of 1016 children died in the first year of life, with 793 being children of Italian parents and 223 being children of  foreign parents. The latest data showed an infant mortality rate of 2.5 per thousand, 2.3 per thousand for children of Italian parents and 3.7 per thousand for children of foreign parents. The MRR of foreigners/Italians, equal to 1.6, decreased compared to the previous year, as the mortality rate for Italian children increased slightly from 2019, while the rate for foreign children decreased (Fig. 3).

In 2020, neonatal mortality in the South was about 70% higher than in the North (2.34 vs. 1.35 per thousand; MRR: 1.74). This territorial inequality was observed both among Italians (2.24 vs. 1.14 per thousand) and among immigrants (3.97 vs. 2.10 per thousand).

In the same period, the difference in post-neonatal mortality between the South and the North was smaller than for neonatal mortality. This applies to both Italians and foreigners: territorial inequality was more evident in the neonatal period than in the subsequent months.

Infant mortality data showed a North–South gradient similar to the neonatal mortality (Table 1).

**Table 1:** Neonatal, post-neonatal and infant mortality rates (NMR, PMR and IMR) per thousand live births according to residence and citizenship. Year 2020

	**NMR**		**PMR**		**IMR**	
	Italians	Foreigners	Italians	Foreigners	Italians	Foreigners
North	1,14	2,10	0,62	1,17	1,77	3,28
Center	1,42	2,91	0,54	1,12	1,96	4,04
South	2,24	3,97	0,78	1,47	3,02	5,44
**Italy**	**1,63**	**2,53**	**0,67**	**1,20**	**2,30**	**3,73**

**Fig. 4:**
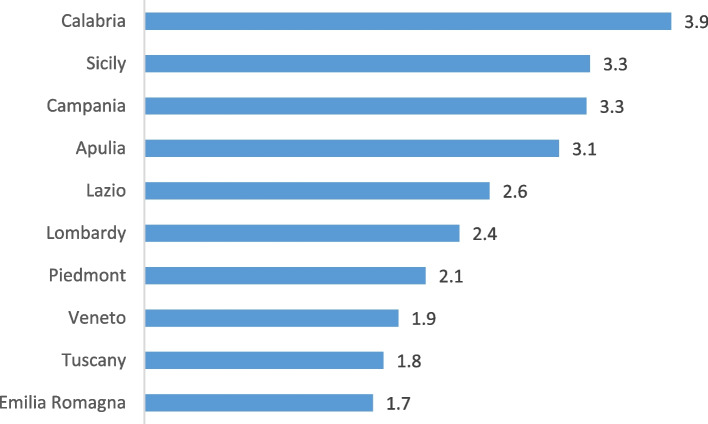
Infant mortality rate by region of residence, per thousand live births

Figure 4 presents infant mortality rates for regions with more than 10,000 live births. The regions with higher mortality rates were those in the South: Calabria, Sicily, Campania, and Apulia. Regions with lower infant mortality rates included Emilia-Romagna, Tuscany, Veneto, and Piedmont. The difference would have been even greater if regions had the same composition of Italians and foreigners.

As seen in Table 2, in northern regions, foreign-born children, who generally had a higher infant mortality rate than Italians, were more numerous. In Emilia-Romagna, Lombardy, Veneto, Tuscany, and Piedmont, the percentage of foreign-born children varied from 19.2% to 24.5%, while in the South (Calabria, Sicily, Campania, and Apulia) it varied from 5.2% to 6.3%. Different regional profiles can be observed regarding the two components of infant mortality, neonatal and post-neonatal.

**Table 2:** Live births, infant mortality rate, percentage of foreign births and deaths, mortality rate ratio (MRR, CI95%) in regions with more than 10,000 live births. Year 2020

Region	Number of live births	Infant mortality rate per 1000 live births			% foreign births /total births	% foreign deaths /total deaths	MRR (CI95%)(foreigners/Italians)
		Italians	Foreigners	Total			
**Lombardia**	69,235	2,0	3,5	2,4	22	32,8	1,75 (1,23; 2,36)
**Campania**	45,078	3,2	5,5	3,3	5,2	8,7	1,72 (0,96; 3,01)
**Lazio**	37,982	2,2	4,9	2,6	15,2	28,5	2,23 (1,42; 3,41)
**Sicily**	37,520	3,3	4,5	3,3	5,3	7,3	1,36 (0,71; 2,75)
**Veneto**	32,672	1,5	3,4	1,9	20,7	36,9	2,27 (1,31; 3,69)
**Emilia Romagna**	29,861	1,3	3,0	1,7	24,5	42,9	2,31 (1,32; 4,00)
**Piedmont**	27,107	1,9	2,8	2,1	19,2	25,3	1,47 (0,77; 2,54)
**Apulia**	26,455	2,9	7,3	3,1	5,6	13,1	2,52 (1,37; 4,94)
**Toscana**	22,380	1,4	3,3	1,8	19,3	35,2	2,36 (1,2; 4,40)
**Calabria**	13,966	3,7	7,8	3,9	6,3	12,5	2,11 (0,94; 4,67)
**Italy**	**404,892**	**2,3**	**3,7**	**2,5**	**15**	**22**	**1,61 (1,38; 1,86)**

**Fig. 5:**
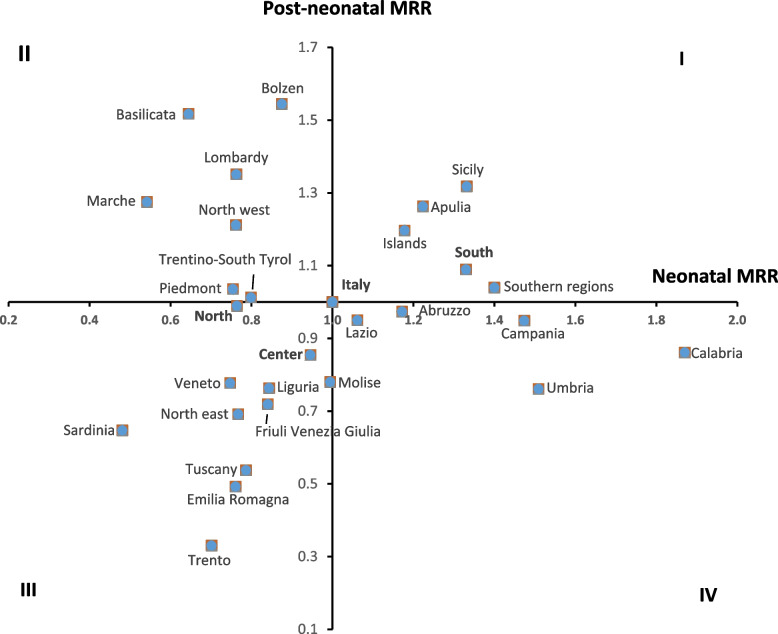
Neonatal (x-axis) and post-neonatal (y-axis) mortality rate ratio (MRR) for regions and geographical areas. *MRRs are calculated by comparing region and area rates to the national ones

Figure 5 shows the ratios between neonatal mortality rates (X-axis) and post-neonatal mortality rates (Y-axis) for each region compared to the national average. It is interesting to note that some regions had only one component higher than the national average, indicating the need for differentiated policies in the area.

Regions in the first quadrant had both neonatal and post-neonatal mortality rates higher than the national average, while regions in the third quadrant had both lower neonatal and post-neonatal mortality rates than the national average. Regions in the second quadrant had only post-neonatal mortality rates higher than the national average, and regions in the fourth quadrant had only neonatal mortality rates higher than the national average.

The most critical situations were observed in Sicily and Apulia, where both neonatal and post-neonatal mortality rates were higher than the national average. Calabria, Umbria, Campania, Abruzzo, and Lazio had neonatal mortality rates above the national average, while their post-neonatal mortality rates were below the average.

On the other hand, the Autonomous Province of Bolzen, Basilicata, Lombardy, Marche, and Piedmont had post-neonatal mortality rates above the national average, while conversely, they had neonatal mortality rates below the average value. A better situation was observed in the Autonomous Province of Trento, followed by Emilia Romagna, Tuscany, Sardinia, Friuli Venezia Giulia, Veneto, and Liguria, in which both neonatal and post-neonatal mortality rates were below the average value.

In 2020, the main causes of death were neonatal respiratory distress (127 deaths) and congenital malformations of the circulatory system (105 deaths). While the latter remained constant compared to the 2016–2019 period, neonatal respiratory distress increased by about 50% (Additional file 1).

To study the impact of variables such as place of residence, citizenship, and calendar year on the risk of death from a specific cause, we employed a Poisson log-linear regression model [11].

**Fig. 6:**
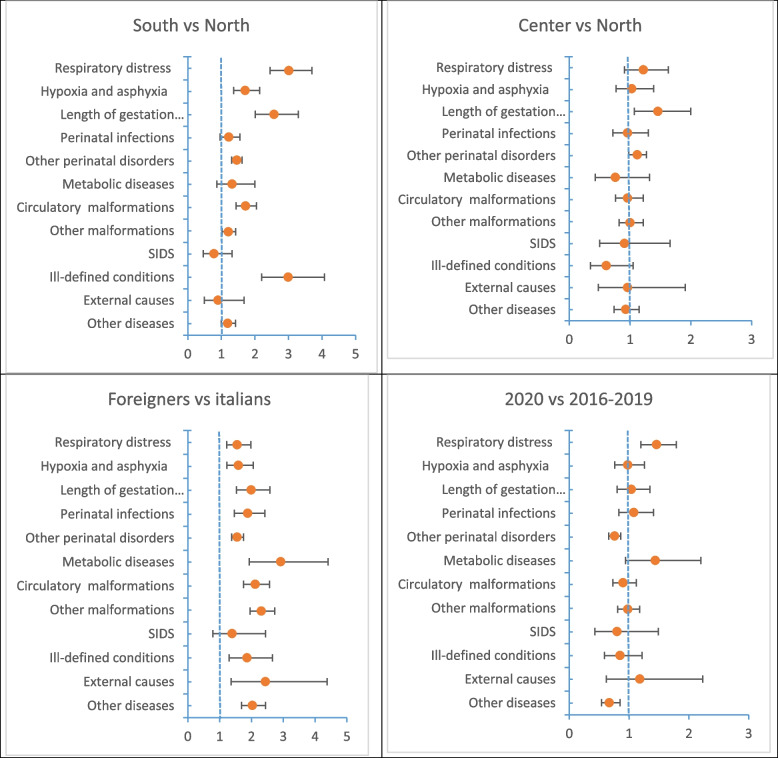
Mortality rate ratios (MRRs, CI95%) for the leading causes of death

The results are reported in Fig. 6 (MRR and CI95%) and Additional file 2.

The most significant results from this analysis are as follows: in South Italy, there was a higher risk of death due to neonatal respiratory distress (MRR: 3.01), premature births (MRR: 2.57), and hypoxia (MRR: 1.71) compared to Northern Italy. There were no significant differences between the Center and the North, except for premature births (MRR: 1.46). Foreign-born children had a higher risk of death for almost all causes of death indicated in Fig. 6.

The highest MRRs were for metabolic (MRR: 2.92), trauma (MRR: 2.44), and other congenital malformations and deformations (MRR: 2.31). The only condition that significantly increased in 2020 compared to the 2016–2019 period was neonatal respiratory distress (MRR: 1.46).

### COVID and infant mortality

From the analysis of individual data in 2020, no child was reported to have died from Covid as the primary cause below one year of age. However, there were two deaths in which Covid-19 was a contributing cause. Therefore, while the SARS-CoV-2 virus did not have a direct effect on infant mortality as it did in other age groups, it is hypothesized to have had an indirect effect on other causes of death. For example, as seen earlier, neonatal respiratory distress increased by 46% in 2020 compared to the four-year average. To better understand what happened, mortality rates for both 2020 and the 2016–2019 period were analysed by quarter, particularly comparing the rates for the two Covid-19 waves (March–May and October-December) of 2020 with those for the June–August quarter of the same year, calculating MRRs and confidence intervals. A similar analysis was conducted for the 2016–2019 period (Tables 3 and 4).

**Table 3:** Infant mortality rate ratio (MRR, CI95%) for quarter, area and year

		MRR (IC95%)	
Year	Area	March–May/June–August	October-December/June–August
2016–2019	North	1,09 (0,96;2,97)	1,12 (0,99;3,04)
	Center	0,97 (0,8;2,7)	0,91 (0,75;2,56)
	South	0,97 (0,86;2,67)	0,88 (0,78;2,46)
	Italy	1,01 (0,93;2,75)	0,97 (0,9;2,65)
2020	North	1,16 (0,87;3,18)	1,05 (0,78;2,93)
	Center	1,27 (0,84;3,5)	0,83 (0,53;2,68)
	South	1,19 (0,92;3,25)	0,95 (0,73;2,7)
	Italy	1,18 (1;3,22)	0,97 (0,81;2,69)

**Table 4:** Neonatal respiratory distress mortality rate ratio (MRR, CI95%) for quarter, area and year

		MRR (IC95%)	
Year	Area	March–May/June–August	October-December/June–August
2016–2019	North	1,47(0,82;4,07)	1,68(0,96;4,56)
	Center	0,89(0,46;3,07)	0,26(0,1;2,95)
	South	1,08(0,76;3,02)	0,58(0,39;2,19)
	Italy	1,1(0,84;3,04)	0,73(0,54;2,3)
2020	North	4,71(1,34;13,41)	3,18(0,86;8,74)
	Center	1,11(0,36;4,44)	0,36(0,07;5,33)
	South	1,41(0,76;3,97)	1,27(0,68;3,68)
	Italy	1,69(1,04;4,6)	1,29(0,78;3,62)

In Table 3, it is evident that in 2020, the Mortality Rate Ratios (MRRs) for infant mortality rates during March–May (1st wave) consistently exceed 1 in all areas, in contrast to the preceding four-year period, even though these differences were not statistically significant.

When examining infant mortality rates due to neonatal respiratory distress and calculating MRRs, they consistently exceed 1 in 2020, with statistical significance observed in the North and in Italy (Table 4) during the March–May period, which marked the onset of the Covid-19 pandemic.

## Discussion

Italy has one of the lowest rates of neonatal and infant mortality in Europe. In 2020, the infant mortality rate (EU27) was 3.3 deaths per thousand live births. Lower values than the Italian rate were observed only in some Northern European countries such as Sweden, Finland, and Norway [12].

This study confirms the higher risk of death for infants born to foreign parents and those born in the South of Italy, as previously reported in a prior study [7].

Immigration in Italy is an important phenomenon that has progressively increased over the past two decades. As of January 1, 2020, the resident foreign population numbered 5,306,548 individuals, accounting for 8.8% of the total residents [13], and it has significantly contributed to the birth rate and helped to make the demographic pyramid less imbalanced. The reasons for higher child mortality among foreign parents can be linked to perinatal conditions that begin before birth, particularly concerning the health of pregnant women. Foreign women are, on average, younger than Italian women. According to the analysis of births in 2020, the average age of mothers at childbirth was 32.7 years for Italians and 29.3 years for foreign citizens [14]. However, even though foreign mothers are younger, and theoretically at lower risk, for a variety of reasons related to social, economic, and cultural disadvantages, such as a higher number of teenage pregnancies and young mothers, low family income, less secure and more demanding employment, inadequate nutrition, poor living conditions, delayed or inadequate obstetric care, they more frequently experience preterm births and perinatal pathologies that affect the newborn [15–17].

In Italy, the National Health Service provides free and guaranteed medical care during pregnancy and childbirth, as well as access to emergency services for all women, even those without residency permits. The higher neonatal and infant mortality among foreigners is primarily due to more frequent perinatal pathologies, insufficient integration of foreign women, lack of information about available services, language difficulties, fear of local authorities, and cultural differences. There is no denying that giving birth far from one's home country, separated from family, in often precarious living and working conditions, with a lack of psychological and emotional support, in a different climate with unfamiliar food, where many things are not understood and much is unknown, objectively constitutes a disadvantaged situation. It is important to facilitate access to healthcare facilities, especially for recently immigrated women, ensuring that they receive assistance tailored to their needs. Additionally, healthcare providers must be prepared for encounters with diverse cultures.

Significantly, the children of foreign parents had a higher mortality rate than Italian children for all major causes of death, especially metabolic diseases and congenital malformations. One explanation could be the higher frequency of consanguineous marriages [18], which are known to increase the chances of both members of the couple being carriers of the same mutation for a recessive disorder.

As already mentioned, there are geographical differences in mortality between children of Italian and foreign parents. The risk of neonatal and infant mortality is higher in the South compared to the North. A child, whether Italian or foreign, born in the South has a 70% higher risk of dying in the first year of life than one born in the North. If the South had the same mortality rate as the North, there would not have been 155 child deaths.

Analysing infant mortality in regions with high birth rates in 2020, those with the highest rates were Calabria, Sicily, Campania, and Apulia, while those with the lowest rates were Emilia-Romagna, Tuscany, Veneto, and Piedmont. When analysing the Mortality Rate Ratio, the risk of infant mortality in Calabria was about two times higher than in Emilia-Romagna and Tuscany (2.31 and 2.20, respectively), and slightly lower for Sicily (1.95 and 1.86). These territorial inequalities would have been even higher if the South had the same presence of foreigners as in Northern Italy. Foreigners have higher mortality rates than Italians, but their presence in the South is much lower than in the North. Being born to a foreigner in the South represents an additional risk. If foreign-born individuals in the Southern regions had the same mortality rate as foreign-born individuals in the North, 17 children would not have died.

Most of the infant mortality in Calabria and Campania occurs in the neonatal period because post-neonatal mortality rates are in line with the national average. This data highlights the importance of giving greater attention to the birth event and preventing situations that could jeopardize the care of both mother and baby. It further confirms the need to improve the maternal and child healthcare network and the organization of birthing centers. The increase in post-neonatal mortality in some regions with better healthcare organizations (e.g., the North West) might be associated with the mortality of children with complex and incurable conditions beyond the neonatal period.

The higher neonatal and infant mortality in the South is mainly associated with historical factors related to economic and social problems, which have worsened in recent years due to the economic crisis that began in 2008 and the COVID-19 pandemic, which has also affected the pediatric population.

It has been observed that children and adolescents living in the South of Italy are at a higher risk of absolute poverty (families and individuals who cannot afford the minimum expenses required for an acceptable standard of living) [19]. This risk is especially pronounced among children with non-Italian parents. 30% of families with only foreign citizens are in absolute poverty, compared to 5.7% of families with only Italian citizens. It is well-known that there is a strong relationship between low socioeconomic status and the risk of illness [20].

Additionally, healthcare organization in the South is undoubtedly less effective than in central and northern regions. An indirect sign of this is the higher healthcare migration of minors from the South to other areas of the country [21].

One factor that has significantly affected the overall level of healthcare assistance is the introduction of economic deficit recovery plans, which have involved most Southern regions. A recent study on the entire population has shown that these recovery plans were effective in reducing the deficit, but they led to a 3% increase in preventable deaths, a decrease in hospital admissions, and an increase in South-North migration [22].

The highest rates of infant and neonatal mortality, observed in the southern regions and among the children of immigrant parents, should prompt policymakers to prioritize maternal and child care. It is urgent to heed the recommendations of the Italian Society of Pediatrics regarding the reorganization of pediatric care [23].

One of the recommended interventions is to reduce the number of small maternity facilities that often lack qualified healthcare personnel to handle emergency situations for both mothers and infants. The shortage of pediatric intensive care beds, particularly severe in the southern regions, must be addressed by increasing their numbers [24]. Additionally, improving the integration of care between hospitals and the community is essential.

Equally important are interventions to address poverty and provide special support to pregnant immigrant women, both before and after childbirth. This includes integration programs featuring multilingual educational pre-birth courses, the establishment of social support networks, and the provision of culturally sensitive post-natal care that respects the cultural background, beliefs, and practices of immigrant mothers. The data collected did not indicate cases of COVID-related mortality, but only two cases where COVID was a contributing disease to the death. This is not surprising because the vertical transmission of the infection from mother to newborn is exceptionally rare. Although neonates can be infected with the SARS-CoV-2 virus through saliva droplets in the post-natal period when they come into contact with infected mothers or healthcare workers, cases of SARS-CoV-2 infection in neonates are rare. Moreover, most infected neonates are asymptomatic or exhibit mild illnesses that do not require respiratory support.

The milder severity of SARS-CoV-2 infection in the early stages of life has been attributed to a more efficient immune system, cytokine production, and lower penetration of the virus into the body's cells [25]. Severe forms that require mechanical ventilation have been described but are very rare. The two children who died with COVID-19 already had severe illnesses.

The increase in infant mortality during the first wave of the pandemic might be associated with inadequate or reduced prenatal care during the lockdown period and/or a lack of timely healthcare assistance.

Regarding the causes of death, a significant increase in mortality due to respiratory distress was observed in 2020, especially in the North. Unfortunately, this study did not analyse deaths based on gestational age, and therefore could not evaluate deaths of preterm infants who are known to be more prone to respiratory problems compared to full-term births. However, some analyses conducted in numerous Italian regions did not show an increase in prematurity during the period 2020–2021[26]. Therefore, it is likely that many women, particularly during the first lockdown period from March to May 2020, did not attend regular prenatal check-ups at the hospital out of fear of contagion, resulting in a higher number of perinatal issues. The most critical period was during the lockdown, especially from March to May 2020. The overall increase in mortality due to respiratory distress in Italy (from 0.21 to 0.31 per thousand live births) mainly affected the North, which was the first area hit by the pandemic. During the first wave, mortality from respiratory distress in these regions doubled compared to the 2016–2019 period, increasing from 0.14 to 0.29 per thousand live births. It was also four times higher than the June–August period of the same year. However, this last data, calculated on a quarterly basis, could be influenced by the limited number of observations. The confidence interval extremes of the MRR calculated by comparing the mortality rate for respiratory distress in the March–May period with that in the June–August period, although significant, are wide (Table 4).

The National Health Service (NHS), introduced with health reform law no. 833 in 1978, based on the principles of universality, equality, and equity, has undoubtedly contributed to improving the health of Italians and has significantly reduced infant mortality in Italy. However, the NHS has faced challenges, which have worsened with progressive funding cuts made by successive governments in recent years and have become more evident, sometimes dramatically, with the COVID-19 pandemic. Investing in children's health, especially in the first one thousand days of life (270 days of pregnancy and the first 2 years), is the most effective means of ensuring healthy adults with a longer and healthier life expectancy.

## Conclusions

While Italy boasts one of the lowest infant mortality rates in Europe and worldwide, our study has revealed hidden disparities, both in terms of geography and ethnicity. The neonatal and infant mortality rates indicate the persistence of profound inequalities in Italy between the North and the South and between Italian and foreign children.

Moreover, the COVID-19 pandemic has indirectly impacted infant mortality, particularly during the first wave (March–May 2020). Geographic disparities in healthcare must be reduced, and equal access to care must be guaranteed for all citizens through the integration programs and the creation of pre-natal and post-natal services that are currently distributed unequally across the territory. It should be noted that in order to improve public health, it is essential to enhance social conditions, and combating poverty should be a priority for every government [19].

### Supplementary Information

**Additional file 1.**

**Additional file 2.** 

## Data Availability

The datasets generated during and/or analyzed during the current study are available from the corresponding author on reasonable request.

## Abbreviations

Istat: Italian National Institute of Statistics
NMR: Neonatal mortality rate
PMR: Post-neonatal mortality rate
IMR: Infant mortality rate
MRR: Mortality rate ratio
